# Antimicrobial susceptibility and genomic characterization of *Lactococcus formosensis*, *Lactococcus garvieae*, and *Lactococcus petauri* in Hong Kong

**DOI:** 10.1128/spectrum.00101-25

**Published:** 2025-08-13

**Authors:** You-Xiang Chan, Huiluo Cao, Chong-Yee Yau, Xin Li, Kin-Hung Chow, Pak-Leung Ho

**Affiliations:** 1 Department of Microbiology, University of Hong Kong, Hong Kong, China; 2 Department of Clinical Pathology, Pamela Youde Nethersole Eastern Hospital, Hospital Authority, Hong Kong, China; 3 Microbiology Division, Department of Health, Public Health Laboratory Services Branch, Centre for Health Protection, Hong Kong SAR, China; 4 Carol Yu Centre for Infection, University of Hong Kong, Hong Kong, China; 5 Department of Microbiology, Queen Mary Hospital, Hospital Authority, Hong Kong, China; Taichung Veterans General Hospital, Taichung, Taiwan

**Keywords:** molecular epidemiology, microbial genomics, resistance genes, sucrose operon

## Abstract

Recent studies have shown that many isolates previously classified as *Lactococcus garvieae* were *L. petauri* and *L. formosensis*, complicating the understanding of the ecological niches, phylogeny, and phenotypic characteristics of these organisms. In this study, a total of 94 human feces and 166 food samples, collected from Hong Kong in 2021–2023, were cultured using selective media, with a positive rate of 21% for human feces, 37% for sashimi, 8% for cut fruits, and 8% for salad vegetables. The isolates (*n* = 67, including 43 from sashimi and four from ready-to-eat cut fruits and vegetables) from the cultured samples and additional human clinical isolates (*n* = 9) were further tested. Among the three species, the antimicrobial resistance profiles were similar, with resistance being extremely high for rifampicin (100%) and clindamycin (71%–92%), very high for cotrimoxazole (53%–58%), moderate to very high for minocycline (12%–56%) and low for levofloxacin (0%–2%). All isolates were susceptible to ampicillin, ceftaroline, daptomycin, erythromycin, linezolid, meropenem, and vancomycin. Phylogenetic analysis revealed several distinct genomic clusters shared by the *L. petauri* isolates from both human and sashimi sources, while no such cross-sectoral clustering was observed for the other two species. The ability to ferment sucrose was linked to the presence of sucrose operons in the isolates, with all *L. petauri* and two *L. garvieae* isolates from Hong Kong exhibiting this trait. In conclusion, this work provides information on the resistance patterns of the three species and evidence of a link among *L. petauri* isolates from human and food sources.

**IMPORTANCE:**

Recent studies have revealed evidence of human and animal host specificity among three closely related *Lactococcus* species: *L. formosensis*, *L. garvieae,* and *L. petauri*. Among the three species, *L. petauri* is the most frequent human pathogen. However, our understanding of the presence of these three species in food samples, which could be potential sources of human infection, remains limited. It is also not clear whether these species display varying susceptibilities to commonly used antibiotics for treating human infection. This study found the frequent contamination of sashimi (37%) with *L. petauri*. Genomic investigation revealed cross-sectoral phylogenetic clustering of some *L. petauri* isolates from sashimi and human sources, indicating a potential mode of human transmission through contaminated sashimi. No cross-sectoral clustering of isolates was observed for the other two species. There are potential risks associated with consuming ready-to-eat foods contaminated with potential pathogens, which can cause health consequences.

## INTRODUCTION

*Lactococcus garvieae* is a species of Gram-positive bacteria that belongs to the lactic acid group. It was identified in 1983 and is recognized for its involvement in lactococcosis in fish and mastitis in cattle, posing a significant threat to aquaculture and livestock industries (1, 2). *L. garvieae* has a wide distribution and has been isolated from different aquatic and terrestrial animals, from river and sewage waters, from many food and feedstuffs (3). In humans, *L. garvieae* is considered an opportunistic pathogen that causes a variety of infections, particularly in those with underlying health conditions or immunosuppression (4). In recent years, human infection by *L. garvieae* appears to be increasing, likely because of improvement in methods for bacterial identification and higher awareness among clinicians (5, 6). However, our understanding of *L. garvieae* is complicated by the recent discovery of two newly identified bacteria, *L. formosensis* and *L. petauri*, which share close genetic relationships with *L. garvieae* (7, 8). Genomic sequencing has revealed that the misclassification of *L. petauri* and *L. formonensis* as *L. garvieae* is prevalent in the scientific literature, indicating that certain infections attributed to *L. garvieae* may be caused by these other two bacterial species (6, 9).

Recent work has shown that these three bacteria exhibit distinct distribution patterns across various sources, with *L. petauri* predominantly associated with human infections and rainbow trout, while *L. formosensis* is commonly found in cases of cow mastitis (2, 6, 9). In controlled laboratory challenges, rainbow trout exhibited greater susceptibility to *L. petauri* infection compared to *L. garvieae* and *L. formosensis* (10). Two subspecies of *L. formosensis* have been identified: *L. formosensis* subsp. *bovis*, known to cause bovine mastitis in China and found in human feces, swine, and pork sausage; and *L. formosensis* subsp. *formosensis*, detected in human infections, fish, fermented broccoli, and insects (7, 9). Variations in the prevalence of 14 adherence genes and a bile salt hydrolase have been observed among the three *Lactococcus* species, potentially contributing to host specificity (9).

In microbiology laboratories, matrix-assisted laser desorption/ionization time-of-flight mass spectrometry (MALDI-TOF MS) supplemented by 16S rDNA sequencing is widely used for bacterial identification (11, 12). Since *L. garvieae* but not *L. formonensis* and *L. petauri* are included in commercially available MALDI-TOF MS databases, the latter two organisms would be misidentified as *L. garvieae* (9). 16S rDNA sequencing cannot reliably differentiate the three *Lactococcus* species because their 16S rDNA sequences are highly similar (13). Whole-genome sequencing (WGS) followed by average nucleotide identity (ANI) and digital DNA-DNA hybridization (dDDH) analysis are reference methods for bacterial identification, but accessibility is limited in many microbiology laboratories (9). Recently, it was suggested that sucrose fermentation could serve as a potential parameter for distinguishing between *L. garvieae* (sucrose-negative) and *L. petauri* (sucrose-positive) strains (14, 15). A study focusing on isolates obtained from fish affected by lactococcosis found that all 27 *L*. *petauri* strains were sucrose-positive, whereas all 10 *L. garvieae* strains were sucrose-negative (15). Similarly, in another investigation involving ATCC reference strains and a total of 137 *L*. *petauri* and 40 *L*. *garvieae* strains from different fish farms, all *L. petauri* isolates exhibited sucrose fermentation, while the *L. garvieae* isolates did not (14). An important limitation of both studies is that the investigated isolates were predominantly collected from fish farms in European countries; few isolates from other geographical areas have not been tested, and the closely related species *L. formonensis* was not tested in parallel. Moreover, the sucrose operon may be located on mobile genetic elements, and phenotypic variability may occur because of horizontal gene transfer and gene inactivation (16).

In humans, these *Lactococcus* organisms are likely acquired through the ingestion of contaminated food, entering the bloodstream via the gastrointestinal tract and spreading to infect distant sites (3, 9). However, few studies have investigated the prevalence of these organisms in food products and the genomic relationship between isolates from food and human sectors (17–19). This study aimed to assess the prevalence of *L. petauri*, *L. garvieae*, and *L. formosensis* in ready-to-eat cut fruits, salad vegetables, sashimi, and human fecal samples collected in Hong Kong. The susceptibility of the isolates to antibiotics commonly used for treating human infection was assessed. Whole genome sequencing and phylogenetic analysis were conducted to investigate the hypothesis of cross-sectoral transmission of isolates from contaminated sashimi and other food items to humans. Additionally, we analyzed the isolates and publicly available genomes to evaluate the utility of sucrose as a test for species differentiation, considering the potential limitations posed by the deletion and acquisition of sucrose operons.

## RESULTS

### Isolation of strains in this study

A total of 67 isolates were recovered from 260 samples (Table 1). These included 43 isolates from sashimi, 20 isolates from human feces, and 2 each from cut fruits and salad vegetables. Each isolate originated from a different sample. The culture positivity rate was 21% for human feces and 8%–37% for the food samples. Culture positivity rate for amberjack, flounder, prawn, and salmon sashimi samples was 34% (11/32), 31% (9/29), 41% (12/29), and 41% (11/27), respectively (chi-square test, *P* = 0.816). All the isolates were identified as *L. garvieae* by MALDI-TOF-MS. The 67 isolates obtained in this study and an additional 9 isolates from human blood cultures were further investigated.

**TABLE 1:** Detection of *Lactococcus formosensis*, *Lactococcus garvieae*, and *Lactococcus petauri* in human and non-human samples, Hong Kong, 2021–2023

	Human feces	Cut fruits	Salad vegetables	Sashimi	Total
Number tested	94	25	24	117^*a*^	260
Number positive	20	2	2	43	67
Culture positivity (%)	21	8	8	37^*b*^	26
MALDI-TOF-MS species					
*L. garvieae*	20	2	2	43	67
WGS species					
*L. formosensis*	0	0	0	6	6
*L. garvieae*	3	1	0	11	15
*L. petauri*	17	1	2	26	46

^*a*^ Including 32 amberjack, 29 flounder, 29 prawn, and 27 salmon samples.

^*b*^ Culture positivity for amberjack, flounder, prawn, and salmon samples was 34% (11/32), 31% (9/29), 41% (12/29), and 41% (11/27), respectively (chi-square test, *P* = 0.816).

### Species identification and antimicrobial susceptibilities

The proportion of isolates with 100% and ≥98% completeness was 75% and 100%, respectively (Table S1). The species assignments of the 76 isolates in the data set were obtained using GTDB-Tk, fast ANI, and dDDH. GTDB-Tk identified 52 as *L. petauri*, 17 as *L. garvieae,* and 7 as *L. formosensis* (Table 1). Identical species results were obtained using fast ANI and dDDH. Among the seven *L*. *formosensis* isolates, two had higher dDDH values with type strain LMG 30663 (85.7%–85.8%) than with type strain NBRC 109475 (81.9%–82.3%) and were identified as *L. formosensis* subsp. *bovis*, whereas five isolates had higher dDDH values with NBRC 109475 (84.9%–86.5%) than LMG 30663 (74.4%–75.3%) and were identified as *L. formosensis* subsp. *formosensis*. In human feces and sashimi samples, *L. petauri* is the predominant species, comprising 85% and 60%, respectively, of the isolates (Table 1). The number of isolates obtained from cut fruits and salad vegetables was small, and thus, no inference could be drawn about the predominant species in these samples.

Among the three species, resistance was extremely high for rifampicin (100%) and clindamycin (71%–92%, *P* = 0.236), very high for cotrimoxazole (53%–58%, *P* = 0.942), moderate to very high for minocycline (12%–56%, *P* = 0.005), and low for levofloxacin (0%–2%, *P* = 0.791). Multidrug resistance (i.e., co-resistance to clindamycin, cotrimoxazole, and minocycline) was moderate to high (12%–27%, *P* = 0.371, Table 2). The resistance rate for clindamycin, cotrimoxazole, levofloxacin, minocycline, as well as multidrug resistance, was not significantly different between human and non-human sources. All isolates were susceptible to ampicillin, ceftaroline, daptomycin, erythromycin, linezolid, meropenem, and vancomycin. The relationship between resistance rate and the two variables, namely species (*L. formosensis*, *L. garvieae*, *L. petauri*) and isolate source (human, non-human), was further assessed using multivariate logistic regression. Minocycline resistance was significantly associated with species (higher in *L. petauri* than the other two species, *P* = 0.014) but not with the isolate source. Resistance rates for clindamycin, cotrimoxazole, levofloxacin, and multidrug resistance were not significantly different by species and source (all *P* value > 0.05).

**TABLE 2:** Antimicrobial susceptibility of *Lactococcus formosensis*, *Lactococcus garvieae*, and *Lactococcus petauri* in this study^*d*^^,^^*e*^

	No. tested	Ampicillin	Ceftaroline	Clindamycin	Cotrimoxazole	Daptomycin	Erythromycin	Levofloxacin	Linezolid	Meropenem	Minomycin	Rifampicin	Vancomycin	MDR^*c*^
MIC_50_	76	0.5	≤0.5	≥4	2	≤0.5	≤0.25	1	2	≤0.25	≤2	≥8	0.5	–^e^
MIC_90_	76	1	≤0.5	≥4	≥8	≤0.5	≤0.25	1	2	≤0.25	≥16	≥8	0.5	–
MIC breakpoint, μg/mL		≥2^*a*^	≥2^*b*^	≥1^*a*^	≥4^*a*^	≥2^*b*^	≥1^*a*^	≥4^*a*^	≥8^*b*^	≥0.5^*a*^	≥8^*b*^	≥2^*b*^	≥4^*a*^	–
% Resistant														
By species														
*L. formosensis*	7	0	0	71	57	0	0	0	0	0	29	100	0	14
*L. garvieae*	17	0	0	88	53	0	0	0	0	0	12	100	0	12
*L. petauri*	52	0	0	92	58	0	0	2	0	0	56	100	0	27
By source														
Human	29	0	0	83	48	0	0	3	0	0	55	100	0	28
Non-human	47	0	0	94	62	0	0	0	0	0	36	100	0	19
Total	76	0	0	89	57	0	0	1	0	0	43	100	0	22

^*a*^ CLSI M45, third edition breakpoints for *Lactococcus* spp.

^*b*^ CLSI M100, 34th edition for *Staphylococcus* spp.

^*c*^ Multidrug resistance (MDR) is defined as co-resistance to clindamycin, cotrimoxazole, and minocycline.

^*d*^ The levels of resistance were graded according to the European Food Safety Authority: rare (<0.1%), very low (0.1%–1%), low (1.1%–10%), moderate (11%–20%), high (21%–50%), very high (51%–70%), and extremely high (≥71%). Gray shading indicates very high and extremely high levels of resistance.

^*e*^ –, not applicable.

### Multilocus sequence typing and core genome SNP analysis

The genetic diversity of the isolates in this study was high. The isolates of *L. formosensis*, *L. garvieae*, and *L. petauri* were classified into 6, 16, and 21 multilocus sequence typing (MLST) types, respectively (Table S2). This led to Simpson’s diversity index values of 0.933 (95% confidence interval, 0.805–1.000), 0.933 (0.805–1.000), 0.897 (0.840–0.954), respectively. Within *L. petauri*, four MLST types (ST10, ST29, ST34, ST35) were observed to contain isolates from both human and food sectors (Fig. S1). However, no such overlap was observed for MLST types of *L. formonensis* and *L. garvieae*.

**Fig 1:**
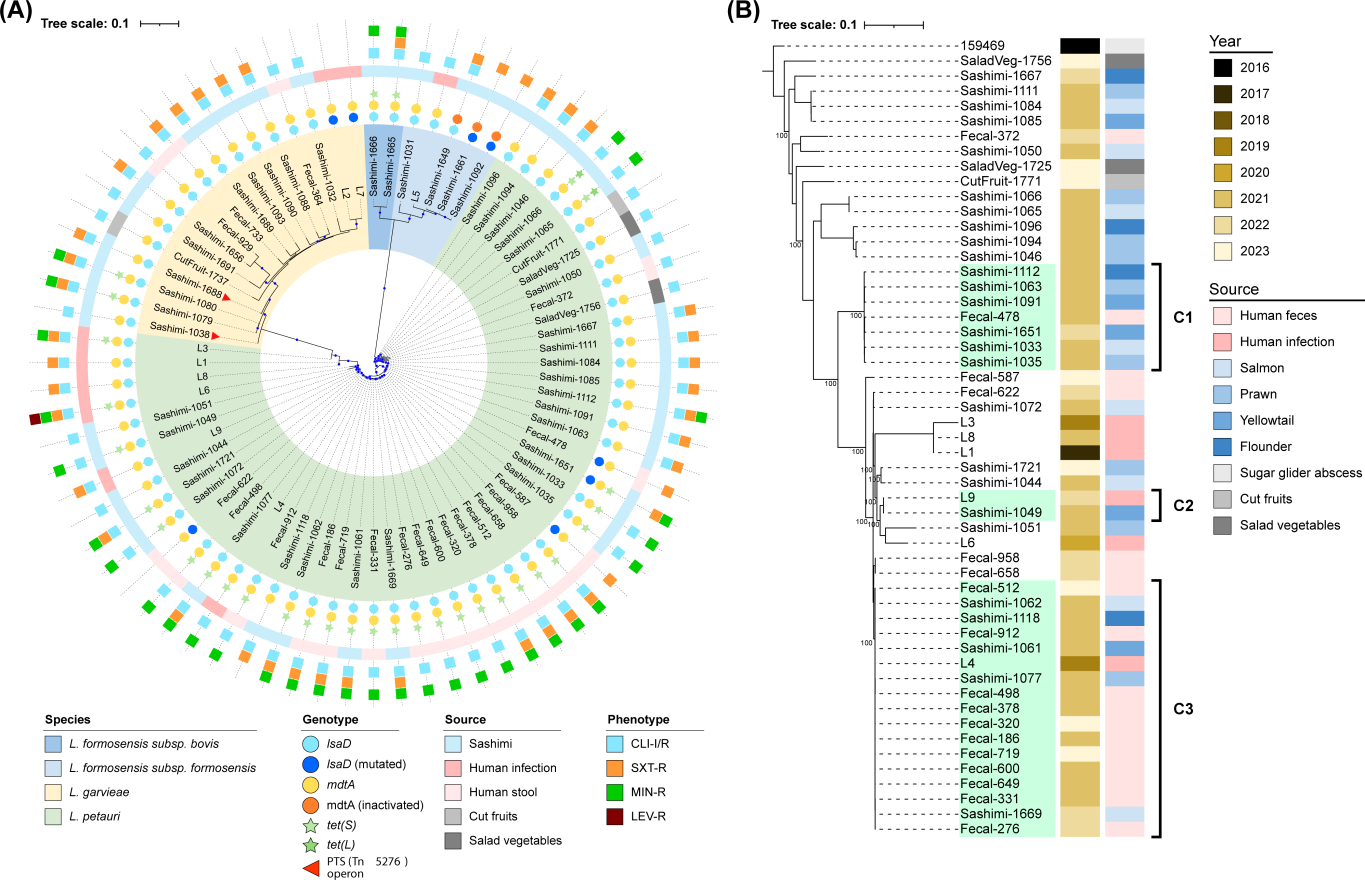
Phylogenies of *Lactococcus formosensis*, *Lactococcus garvieae*, and *Lactococcus petauri* isolates from Hong Kong, 2021–2023. (**A**) Phylogeny for the 76 isolates representing the three species was constructed using 60,516 core SNPs, with the best-fit model (TVM+F+ACS+R3) selected by ModelFinder Plus in IQ-Tree and midpoint rooting. Nodes with strong bootstrap support ≥90% are marked with blue solid circles. From outer to inner circle, the illustration shows the resistance phenotype (CLI-R, clindamycin; SXT-R, cotrimoxazole; MIN-R, minocycline; LEV-R, levofloxacin), isolate source, and resistance genotype. The two sucrose-positive *L. garvieae* isolates were indicated with orange arrows. (**B**) Phylogeny for 53 *L*. *petauri* isolates was built from 72,088 core SNPs using the best-fit model (TVMe+ACS+R2) selected by ModelFinder Plus in IQ-Tree and rooted at the midpoint. Credible bootstrap support values ≥90% for nodes are shown. Three clusters (C1, C2, C3) of cross-sectoral isolates were marked.

The isolates were subjected to core genome single-nucleotide polymorphism (SNP) analysis to further investigate the presence of cross-sectoral clusters between isolates originating from human and food sources (Table S3). The results revealed cross-sectoral clustering among the *L. petauri* isolates, while no such clustering was observed among the *L. formosensis* and *L. garvieae* isolates (Fig. 1A). Within the *L. petauri* isolates, a phylogenetic tree based on 72,088 core SNPs revealed three cross-sectoral clusters (Fig. 1B). These included seven isolates in cluster 1 with 1–202 SNP differences in pairwise comparison, two isolates in cluster 2 with 112 SNP differences in pairwise comparison, and 17 isolates in cluster 3 with 7–137 SNP differences in pairwise comparison (Fig. 1B; Table S4).

### Acquired and chromosomal resistance genes

We grouped the antibiotic resistance genes (ARGs) according to ResFinder (Fig. 1). Two resistance genes, *mdtA* (multidrug resistance efflux protein) and *lsaD* (ribosomal protection protein), were detected in chromosomal assemblies in all 76 isolates (Table S2). One isolate (L2) had an additional copy of *mdtA* on a plasmid contig. In three isolates (Sashimi-1092, -1649, and -1661), the *mdtA* gene was inactivated by a frameshift mutation. The *mdtA* and *lsaD* genes in the chromosomal assemblies were not located within any mobile genetic elements. In 68 isolates with a clindamycin-resistant phenotype, there was an intact *lsaD* gene with wild-type sequences. In eight isolates with clindamycin-susceptible phenotype, disruption in *lsaD* was observed, including internal stop codon in two isolates and truncation by an IS-*LL6* insertion in one isolate, along with substitutions in the Walker B key motif (D447Y in two isolates), the nucleotide binding region (one L355P), and the interdomain linker region (two R246H) (Fig. S2). The presence of an extra copy of *mdtA* (one isolate) and its inactivation (three isolates) did not affect the MIC of clindamycin. One isolate (Sashimi-1649) with intact *lsaD* and Δ*mdtA* was clindamycin-resistant (MIC > 2 µg/mL), while isolates with Δ*lsaD*/Δ*mdtA* (Sashimi-1092 and -1661) and Δ*lsaD*/intact *mdtA* (fecal-622) were not clindamycin-susceptible (MIC ≤ 0.12 µg/mL) (Table S2). In 33 isolates with minocycline-resistant phenotype, tetracycline resistance genes, including *tetS* alone (*n* = 31) or *tetS* plus *tetL* (*n* = 2), were detected within transposon-like structures ranging in size from 3.9 to 6.3 kb, with IS*1216* present on both ends. The 33 Tn*1216*-like structures carrying *tetS* were detected in plasmid (*n* = 14) or chromosomal contigs (*n* = 19). The two Tn*1213* structures carrying *tetL* were detected in plasmid contigs. Additionally, substitutions in the *gyrA* (S83R) and *parC* (S80R) genes were detected in the only levofloxacin-resistant isolate.

### Sucrose operons and sucrose acidification phenotype

**Fig 2:**
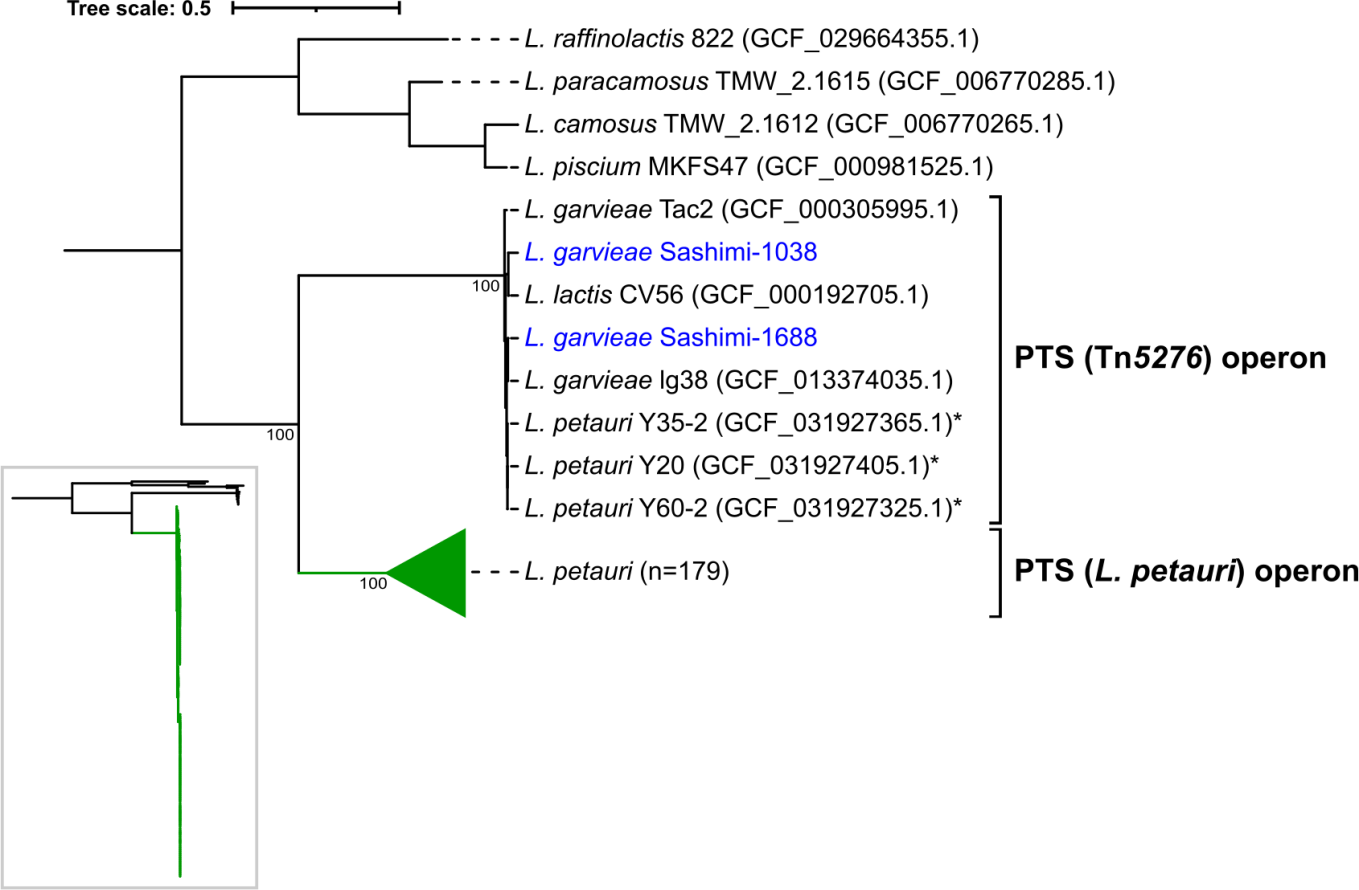
Phylogenetic analysis of PTS operons across seven *Lactococcus* species. The maximum-likelihood tree was built using the HKY+F+I+R3 model selected by ModelFinder Plus in IQ-Tree following MUSCLE alignment and rooted at the midpoint. (Left) The full phylogeny shows evolutionary relationships among all strains. (Right) Focused view highlights the expanded PTS (Tn*5276*) and the collapsed cluster representing 179 conserved PTS (*L. petauri*) operons. Nodes with credible bootstrap support values ≥90% are indicated.

The data set included 317 genomes (Table S1). These included 76 isolates collected in this study and an additional 241 genome assemblies of *L. formonensis*, *L. garvieae,* and *L. petauri* from the National Center for Biotechnology Information (NCBI). All species were ascertained using GTDB-Tk. In these organisms, the phosphotransferase system (PTS) sucrose operon consists of four genes, including a sucrose operon repressor (*sacR*), a sucrose-6-phosphate hydrolase (*sacA*), a beta-glucoside transporter (*sacB*), and a fructokinase (*sacK*). Two sequence types of PTS sucrose operons, including PTS (Tn*5276*) and PTS (*L. petauri*), were detected. The PTS (Tn*5276*) operon was detected in four *L*. *garvieae* (including sashimi-1038 and sashimi-1688 from Hong Kong, Tac2 from turkey meat in Italy, and lg38 from a tofu sample in Japan) and three *L*. *petauri* (Y20, Y35-2, Y60-2 from fish samples in mainland China) isolates (Fig. 2). The 7 PTS (Tn*5276*) operons clustered with the reference operon in *Lactococcus lactis* CV56 at >98% nucleotide identities (Table S5). The PTS (*L. petauri*) operon was identified in 179 out of 182 *L*. *petauri* isolates, with these sequences sharing >98% nucleotide identity with the one in the type strain *L. petauri* 159469. The nucleotide homology between the PTS (Tn*5276*) and PTS (*L. petauri*) operon sequences was approximately 70% (Fig. 2). Three *L. petauri* isolates from mainland China (Y35-2, Y60-2, Y20) possessed both the PTS (*L. petauri*) and PTS (Tn*5276*) operons (Fig. S3). The non-phosphotransferase system (NPTS) operon, consisting of a sucrose operon repressor (*scrR*), a fructokinase (*scrK*), a sucrose-6-phosphate hydrolase (*scrB*), and a lactose permease (*lacY*), was identified in two isolates: *L. garvieae* BA06/02552-1 from a water sample in Spain and *L. petauri* FDAARGOS_1063 from bovine mastitis. In the two isolates, the NPTS operon shared around 96% nucleotide identity (Fig. S4). In the *L. petauri* FDAARGOS_1063 genome, it was situated within a 25.9 kb region bordered by IS-*LL6* on both ends. In *L. garvieae* BA06/02552-1, the NPTS operon was identified in a contig with different flanking regions (Fig. S4). In three *L. petauri* isolates from Europe (INF110, 57, and BM06/00349), both PTS and NPTS operons were absent (Fig. S3).

**Fig 3:**
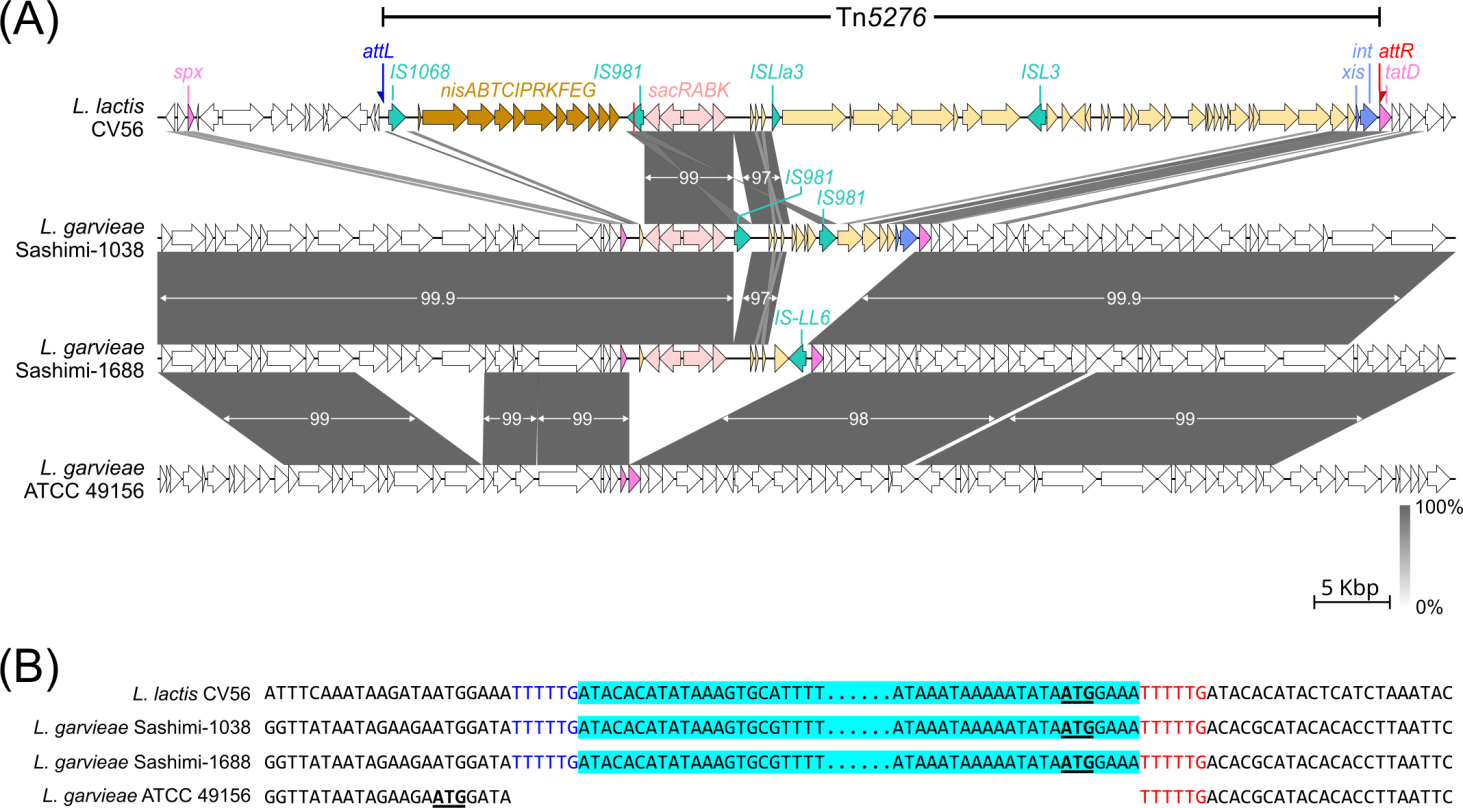
Comparative analysis of sucrose operon-carrying regions in three strains with *L. garvieae* ATCC 49156 as the reference. (**A**) Linear maps showing the insertion sites of sucrose operon-carrying regions in CV56, sashimi-1038, and sashimi-1688. The genes (*spx* and *tatD*) flanking the sucrose operon-carrying regions are indicated in pink. The genes in the PTS sucrose operon (*sacRABK*) are indicated in salmon. (**B**) Nucleotide alignment of Tn*5276* junction regions in CV56 with that of sashimi 1038, sashimi 1688, and ATCC 49156. The sucrose operon-carrying regions (blue highlight), start codon of the *tatD* gene (underlined), *attL* (blue font), and *attR* (red font) sites are indicated in the alignment.

In *L. garvieae* strains sashimi-1038 and -1688, the inserts carrying the PTS (Tn*5276*) sucrose operon had lengths of 19.3 and 12.1 kb, respectively, and were inserted between the *spx* and *tatD* genes in the chromosome (Fig. 3). The *attL* and *attR* sites were similar to those in CV56. Similarly, the PTS (Tn*5276*) operons in the three *L*. *petauri* isolates were detected in inserts between the *spx* and *tatD* genes (Fig. S3). In the *L. petauri* isolates, the PTS (*L. petauri*) operon was detected in the chromosome between the ABC transporter permease gene (GenBank accession CP146754.1, locus tag V7A35_RS01900) and the *rsmA* gene (CP146754.1, locus tag V7A35_RS01925), and no insertion sequence elements or transposons were detected in the adjacent regions.

The 76 isolates from this study were tested phenotypically for sucrose acidification. Consistent sucrose fermentation results were obtained when tested using both the broth and agar methods (Table 3). Among these isolates, the proportion of *L. formosensis*, *L. garvieae,* and *L. petauri* isolates found to ferment sucrose was 0%, 11.8%, and 100%, respectively. In these isolates, the ability to cause sucrose acidification was found to correlate with the presence of the PTS (Tn*5276*) and PTS (*L. petauri*) sucrose operons. Among the isolates from the NCBI, the proportion of *L. formosensis*, *L. garvieae,* and *L. petauri* isolates predicted to ferment sucrose was 0%, 5.7%, and 97.7%, respectively.

**TABLE 3:** Distribution of sucrose fermentation phenotype and sucrose operon among *Lactococcus formosensis*, *Lactococcus garvieae*, and *Lactococcus petauri^f^*

Source and species	*n*	No. (% by row) of sucrose acidification^*a*^		No. with sucrose operon^*d*^		
		Positive	Negative	PTS (Tn*5276*)	PTS (*L. petauri*)	NPTS
This study						
*L. formosensis*	7	0 (0)	7 (100)	0 (0)	0 (0)	0 (0)
*L. garvieae*	17	2 (12)	15 (88)	2 (12)	0 (0)	0 (0)
*L. petauri*	52	52 (100)	0 (0)	0 (0)	52 (100)	0 (0)
NCBI						
*L. formosensis*	58	0 (0)	58 (100)	0 (0)	0 (0)	0 (0)
*L. garvieae*	53	3 (6)^*b*^	50 (94)	2 (4)	0 (0)	1 (2)
*L. petauri*	130	127 (98)	3 (2)^*c*^	3 (2)^*e*^	127 (98)	1 (1)^*e*^

^*a*^ Results of sucrose acidification for isolates from NCBI were obtained by genotypic prediction based on the presence or absence of the sucrose operon in the genomes.

^*b*^ Presence of the PTS operon (Tn*5276*) in two isolates and NPTS operon in one isolate.

^*c*^ Both PTS and NPTS operons were not detected in three isolates.

^*d*^ The genes in the PTS operon included the following: *sacR*, sucrose operon repressor; *sacA*, sucrose-6-phosphate hydrolase; *sacB*, PTS beta-glucoside transporter; *sacK*, fructokinase. The genes in the NPTS sucrose operon included the following: *scrR*, sucrose operon repressor; *scrK*, fructokinase; *scrB*, sucrose-6-phosphate hydrolase; *lacY,* lactose permease.

^*e*^ Two isolates have both the PTS (Tn*5276*) and PTS (*L. petauri*) operons, and one isolate has both the PTS (LP) and NPTS operons.

^*f*^ PTS, phosphotransferase system; NPTS, non-phosphotransferase systems.

## DISCUSSION

This study presents information on the prevalence of three *Lactococcus* species in human carriage, ready-to-eat food items, and sashimi. The positivity rate in human fecal samples is significantly higher than the 1.3% found in a previous study that performed culture by direct plating onto Columbia Nalidixic acid agar plates (19). The higher positivity rate in our work can be attributed to the incorporation of a broth-enriched step, followed by plating onto a more specialized agar medium. The tested samples of cut fruits and salad vegetables were ready-to-eat and had been washed before being displayed for sale. The lower positive rate of *Lactococcus* in cut fruit and salad vegetable samples compared to sashimi could be due to the washing or processing steps carried out prior to sale. Although *L. garvieae* has been found in various food items, research on its prevalence is limited, and methods to distinguish *L. garvieae* from the other two *Lactococcus* species were not utilized (3). In one study that examined 40 food samples bought from supermarkets, *L. garvieae* was identified in 8% of cereals, 31% of vegetables, and 100% of raw meat (17). The high positivity rates in common food items suggest that humans are regularly exposed to these organisms through dietary intake. These organisms have low human pathogenicity but can enter the bloodstream through anatomically or physiologically altered gastrointestinal tracts, spreading to infect distant sites and potentially causing human diseases like infective endocarditis, mycotic aneurysm, and spondylodiscitis (4, 6).

Currently, commercial microbial identification systems based on MALDI-TOF MS systems cannot differentiate *L. garvieae* from *L. formonensis* and *L. petauri*. The success of MALDI-TOF MS in microbial identification is attributed in part to the abundance of ribosomal proteins among the ions in the analyzed mass range (11). Consequently, organisms that pose challenges for differentiation through 16S rRNA sequencing are likely to present similar challenges for MALDI-TOF MS, as ribosomal proteins have evolved in a comparable manner to 16S rRNA sequences (20). Therefore, augmenting the mass spectra library with additional spectra of *L. formonensis* and *L. petauri* may not definitively address this issue. To differentiate closely related species, one effective approach involves developing classification models based on a large number of spectra for the three *Lactococcus* species using tools like ClinProTools (20, 21). Before a reliable method can be implemented, a note can be included in the identification report to inform users about this limitation of MALDI-TOF MS in distinguishing among the three *Lactococcus* species. Misidentification can adversely affect the investigation of *Lactococcus* outbreaks, epidemiological investigation of the source of human infection, and our understanding of the host and tissue tropism of the three *Lactococcus* species (9, 15). For example, *L. garvieae,* reported to cause contamination of platelet concentrates, was re-identified as *L. petauri* (9, 22). In another report, among 39 *L*. *garvieae* isolates from bovine mastitis in China, 38 isolates were re-identified as *L. formosensis* and one as *L. petauri* (2, 9).

The identification of genomic clusters shared by *L. petauri* isolates from both human and food sources aligns with the host preference previously documented by our team (9). These cross-source genomic clusters were supported by phylogenetic analysis in this study, demonstrating SNP differences consistent with our previous research (9). In human fecal samples, *L. petauri* is the predominant species detected, indicating that this particular species may have a greater capacity for gut colonization in humans compared to *L. formonesis* and *L. garvieae*. The finding that cross-sectoral clustering was not found for *L. formosensis* and *L. garvieae* aligns with previous studies that humans are not the preferred host for the two species (2, 9).

Furthermore, our data demonstrated that the three *Lactococcus* spp. are generally susceptible to drugs such as ampicillin, daptomycin, linezolid, and vancomycin that are commonly used to treat human infection (4, 6). Nonetheless, clinicians should be cautioned that all isolates are uniformly resistant to rifampin, as this drug may be part of the treatment regimen for infective endocarditis (5). Consistent with earlier reports, the resistance rate to clindamycin was very high, while that for cotrimoxazole and minocycline ranged from high to very high (2, 23, 24). In *Lactococcus lactis*, the expression of the *mdtA* increased the MIC of lincomycin but not of clindamycin (25). In *L. garvieae*, the role of *lsaD* in clindamycin resistance has been confirmed by functional studies (26). In our isolates, phenotypic resistance to clindamycin correlated with the presence of an intact *lsaD* gene in the three species, while mutational inactivation or certain amino acid substitutions of the *lsaD* gene were associated with clindamycin susceptibility. Substitutions at positions 246 and 447 of LsaD have previously been reported to lead to clindamycin susceptibility, while the substitution at position 355 is a novel polymorphism linked to clindamycin susceptibility in the current study (26, 27). Resistance to minocycline correlated with the acquisition of a plasmid or transposon carrying *tetS* or *tetL* genes, which are prevalent in multiple other Gram-positive bacteria (28–30).

In this study, the genome characteristics of the PTS and NPTS operons in three closely related *Lactococcus* species were compared. The findings suggest that sucrose acidification is not a reliable indicator for distinguishing between the three species. *L. garvieae* can obtain the PTS sucrose operon via a transposable element, resulting in a sucrose-positive trait, while *L. petauri* may become sucrose-negative due to a mutational deletion in the PTS operon. Consequently, there is currently no simple method available to complement MALDI-TOF MS for distinguishing these three species. Saticioglu et al. designed two primer pairs, LP_IBS and LG_IBS, which target an ABC transporter permease gene and a glycosyltransferase gene, respectively (14). Using these primers, all 138 *L*. *petauri* and 43 *L*. *garvieae* isolates were accurately distinguished. Ustaoglu et al. designed two primer pairs for *L. petauri* and *L. garvieae*, targeting a permease component and a DUF1430 domain-containing protein, respectively (31). All 86 *L*. *petauri* and 39 *L*. *garvieae* isolates were correctly distinguished. An unexpected finding is that the primer pair LG_IBS, designed by Saticioglu et al. for *L. garvieae* identification, was found to cross-react and produce false-positive results with *L. petauri* strains (31). These results indicate that false-positive and false-negative results can occur due to strain-related variation in gene sequences, emphasizing the need for the inclusion of isolates from diverse geographic origins and sources. From what we know, no multiplex PCR assays have been developed that can differentiate *L. formonensis* from *L. petauri* and *L. garvieae*.

Sucrose is commonly used as a sweetener in the human diet. The consumption of sugars has been suggested to be linked to changes in the composition of the gut microbiome (32). The presence of a chromosomally encoded sucrose operon in *L. petauri*, but not *L. garvieae* and *L. formosensis*, may potentially contribute to the adaptation of *L. petauri* in the human gut and its association with human infections. This work showed the presence of the PTS (Tn*5276*) operon, initially described in *L. lactis*, in some *L. garvieae* and *L. petauri* isolates. It was previously suggested that the PTS sucrose operon in *Lactococcus* spp. and *Clostridium* spp. may have a common evolutionary origin (33). In the human gut, dietary sucrose could potentially create selective pressure for the horizontal transfer of PTS (Tn*5276*) from *L. lactis* to *L. garvieae* and *L. petauri*. Future studies could investigate whether colonization of the human gut by these *Lactococcus* species is facilitated by acquisition of PTS (Tn*5276*) and the effect of a sucrose-rich diet.

This study has several strengths, including an integrated analysis of isolates from ready-to-eat food and sashimi, along with human disease and carriage isolates, genomic characterization, and correlation of phenotypic and genotypic traits. However, it is important to acknowledge the limitations of the current study. Firstly, the study is constrained by the relatively small number of isolates, especially for *L. formosensis*. Secondly, the human and food samples were collected from a single geographical region. Thirdly, the country of origin for the sashimi and cut fruit samples was not available, limiting the ability to draw inferences between the genomic clusters and geographical origin. Additionally, food types such as cereals and dairy products, which could be contaminated by these *Lactococcus* species, were not sampled. Furthermore, the PTS (*L. petauri*) sucrose operon lacked functional validation, despite its association with sucrose fermentation phenotype and homology to genes in the validated PTS (Tn*5276*) sucrose operon (34). To address these limitations, future studies should consider taking a wider range of food samples from various geographical regions.

### Conclusions

This work documents the frequent contamination of sashimi and certain ready-to-eat foods with *L. formosensis*, *L. garvieae,* and *L. petauri*. There are potential risks of consuming ready-to-eat foods contaminated with potential pathogens, which can cause health consequences. Furthermore, sucrose acidification is not a reliable test for differentiating between *L. garvieae*, *L. formosensis,* and *L. petauri*.

## MATERIALS AND METHODS

### Isolation of strains in this study, identification, and antimicrobial susceptibility

The samples were collected by convenience sampling during October 2021–March 2023. Human feces were collected from those submitted to a hospital laboratory for clinical investigation. All were outpatient samples and were de-identified before testing. Non-human samples, including ready-to-eat cut fruits, salad vegetables, and sashimi, were purchased from local supermarkets (*n* = 20) and Japanese restaurants (*n* = 32) located in 11 different districts in Hong Kong (Table S6). Only specific types of sashimi, including amberjack (*n* = 32), flounder (*n* = 29), prawn (*n* = 29), and salmon (*n* = 27), were purchased for this study. For each sample type, one to nine samples were purchased from each supermarket and restaurant. The salad vegetables included samples originating from Italy, Thailand, and the United States. The country of origin for the majority of cut fruits and sashimi samples was not known, as the information was not labeled at the retail points.

For culture isolation, 25 g of food samples and approximately 0.1 g of fecal material were enriched in 225 mL and 10 mL brain-heart infusion broth supplemented with 2% sodium chloride and colistin (10 µg/mL), respectively, at 37°C for 24 hours. After enrichment, a 10 µL aliquot of each enriched broth was plated onto selective agar plates, including LG agar (containing 50 g sucrose, 30 g Difco Oxgall, 9 g proteose peptone number 3, 6 g pancreatic digest of casein, 5 g proteose peptone, 1 g dextrose, 15 g agar Oxoid agar no. 1 per liter) and chromoID Strepo B (bioMerieux, Marcy l’Etoile, France), and then incubated aerobically at 37°C for 18–24 hours (35, 36). Subsequently, the plates were inspected for presumptive positive results, which were defined as black colonies with red halo on LG agar or teal-colored colonies on chromoID Strepto B agar. From each presumptive positive agar plate, 1 to 3 colonies displaying indicative morphologies were selected for further analysis. Negative controls consisting of un-inoculated broth were included to guard against contamination in the laboratory.

Bacterial identification was performed using MALDI-TOF MS (Bruker Daltonics, Bremen, Germany). Susceptibility of antimicrobial agents was determined by broth microdilution using Sensititre (Thermo Fisher Scientific, East Grinstead, West Sussex, UK) 96-well panels. The tested antimicrobial concentration ranges were as follows: ampicillin, 0.12–8 µg/mL; ceftaroline, 0.5–4 µg/mL; clindamycin, 0.12–2 µg/mL; trimethoprim-sulfamethoxazole (cotrimoxazole), 0.5/9–4/76 µg/mL; daptomycin, 0.5–4 µg/mL; erythromycin, 0.25–4 µg/mL; levofloxacin, 0.25–4 µg/mL; linezolid, 1–8 µg/mL; meropenem, 0.25–2 µg/mL; minocycline, 2–8 µg/mL, and vancomycin, 0.25–32 µg/mL. *Streptococcus pneumoniae* ATCC 49619 was used for quality control. The results were interpreted in accordance with the Clinical and Laboratory Standards Institute (37, 38). The level of microbiological resistance, encompassing intermediate and resistant levels, was graded according to the European Food Safety Authority (39).

### Sucrose fermentation

The capacity of bacterial isolates to ferment sucrose was tested using sucrose broth and agar as growth media (40). The isolates were cultured on blood agar plates, and overnight cultures were utilized as the inoculum. The sucrose broth composition included 10 g bacteriological peptone (Oxoid L37), 5 g sodium chloride, 10 g sucrose, and 10 mL Andrade’s indicator with pH adjusted to 7.5–7.8 per liter. The sucrose agar formulation comprised 15 g bacteriological agar (Oxoid no. 1), 20 g bacteriological peptone (Oxoid L37), 10 g sucrose, and 10 mL Andrade’s indicator per liter. Following sterilization, the broth was dispensed into 5 mL tubes and inoculated with a loopful (1 µL loop) of bacteria. The test organism was streaked onto a sucrose agar plate. Results were read after incubation for 24 hours at 37°C in ambient air. A change in color of the broth from colorless to pink and the growth of pink color colonies on the agar was considered indicative of positive sucrose fermentation. One each day of testing, two clinical isolates of *L. petauri* (sucrose positive) and *L. garvieae* (sucrose negative), and un-inoculated media were included as controls (9).

### Whole-genome sequencing and analyses

The whole-genome sequencing and analyses were performed mainly following our recent publications (9, 41, 42). In brief, genomic DNA was extracted and qualified before sequencing using Illumina NovaSeq or iSeq100 platforms for all strains. Some representative strains were also subjected to the Nanopore MinION platform for hybrid assembly. Solo short reads were assembled using SPAdes v3.15, while hybrid assembles of long and short reads were performed using Unicycler v0.50, before evaluating the quality of all assembles using QUAST v5.0.2 and CheckM v1.2.2 with cutoffs at >95% completeness and <5% contamination (43).

Systematics and genomic diversity of genomes were explored using species delineation methods and phylogenomic analyses. Species delineations were conducted using three tools, including GTDB-Tk, fastANI for ANI (with ≥95%–96% as the cutoff), and dDDH (with ≥70% as the cutoff), as done in our previous work with the same type strains (9). Core genome SNPs of two datasets (76 genomes of species *L. formosensis*, *L. garvieae,* and *L. petauri*; and 53 *L*. *petauri* genomes) were called using ParSNP v1.7.4 with a randomly selected genome as reference and submitted to IQ-TREE v2.3.6 for phylogenomic analysis using the best model selected from ModelFinder Plus (MFP) and 1,000 bootstrap iterations for node support statistics as done before (9). The tree was visualized using iTOL (https://itol.embl.de). A matrix of SNP distance of genomes was calculated from the core genome alignment using snp-dists (https://github.com/tseemann/snp-dists). MLST profiles for all genomes were identified using a mlst tool (https://github.com/tseemann/mlst) with updated profiles sourced from PubMLST (https://pubmlst.org/). The genetic relationships were then analyzed using GrapeTree v1.5.0 (44). The Simpson’s index of diversity (95% confidence interval) of MLST profiles was calculated using an online tool (http://www.comparingpartitions.info/).

Genomes were annotated using the Prokaryotic Genome Annotation Pipeline (PGAP) (45). ARGs were identified with the CARD database 2023 release v3.2.8 using RGI v6.0.3 (46). Chromosomal resistance gene mutations were validated by aligning with reference proteins topoisomerase-encoding genes *gyrA* (WP_004258266.1), *gyrB* (WP_002283665.1), and *parC* (WP_042218931.1) using Muscle v5 (47). For the ARGs, *mdtA*, *lsaD*, *tetS*, and *tetL*, we examined several types of mobile genetic elements, including insertion sequences, prophages, plasmids, integrons, and integrative and conjugative elements (ICEs)/integrative and mobilizable elements within their 5 kb upstream and downstream flanking regions, following a previously established approach (48).

### Detection of sucrose operons

To comprehensively understand the PTS in *Lactococcus* species, the PTS operon (*sacR*, *sacA*, *sacB*, *sacK*) was surveyed in 76 genomes from the present study and 239 genomes retrieved from NCBI and previous publications (accessed on 22 April 2024) (Table S1). The whole PTS operon sequence (coordinates 619,709–625,096 on the chromosome CP002365) of *L. lactis* CV56 (GCF_000192705.1) was extracted as a query to search all genomes mentioned above using BLASTn with an e-value of 1e-4. All hits with coverage over 80% and identity higher than 60% were retrieved from genomes referring to the coordinates on the contigs. The genes *spx* and *tatD*, observed to be located beyond the two ends of PTS, were used as anchors to validate PTS operons in the genomes. Cluster analysis of these hits was done using CD-HIT v4.8.1 with alignment coverage 0.8 and a sequence identity cutoff of 0.9 (49). All candidate PTS operons were aligned with PTS operons of *Lactococcus raffinolactis* 822 (GCF_029664355.1), *Lactococcus paracamosus* TMW_2.1615 (GCF_006770285.1), *Lactococcus camosus* TMW_2.1612 (GCF_006770265.1), and *Lactococcus piscium* MKFS47 (GCF_000981525.1) as out-groups using Muscle v5 with default settings (47). The phylogeny of these PTS operons was built based on the alignment using IQ-TREE with MFP to select the best model and 1,000 ultrafast-bootstrap tests for node support statistics. To illustrate the structure and potential transferability of PTS operons, the representative PTS with flanking conserved regions, including *L. garvieae* strains sashimi-1038 and sashimi-1688 from the present study, were retrieved to align with *L. garvieae* ATCC 49156 (GCF_000269925.1) as reference and visualized using Easyfig v2.2.2 (50). For the PTS operons dwelling in transposon Tn5276, the whole ICE with flanking regions was retrieved for alignment. Another sucrose utilization pathway, NPTS, was screened from all genomes using the key protein sucrose permease LacY (WDA67752) as a query to perform BLASTp with an e-value of 1e-4 (51). The completeness of NPTS was further surveyed by extracting the flanking genes of *lacY* and comparing them with the NPTS of *L. lactis* LB7 (CP117409.1, coordinates 1437062-1441724), which has been reported to carry a complete NPTS (52).

### Statistical methods

Fisher’s exact test or chi-square test was used to compare proportions. The 95% confidence interval of proportions was estimated using an online calculator (MedCalc Software Ltd., Belgium, version 23.0.6; https://www.medcalc.org/; accessed 5 November 2024). Multivariate logistic regression was used to assess the association between species (*L. formosensis*, *L. garvieae*, *L. petauri*), source (human, non-human), and antimicrobial resistance (clindamycin, cotrimoxazole, levofloxacin, minocycline). A *P value* of <0.05 was considered to indicate statistical significance.

## Data Availability

All data are included in the article and its supplemental material. All genome sequences determined in the present study were deposited in GenBank under BioProject PRJNA1074855.

## SUPPLEMENTAL MATERIAL

Figure S1 to S4 spectrum.00101-25-s0001.pdf Figure S1: Visualization of MLST genetic clusters for *L. petauri*, *L. formosensis*, and *L. garvieae* from Hong Kong, 2021-2023. Figure S2: Alignment of LsaD amino acid sequences from 11 isolates. Figure S3: Comparative analysis of sucrose operon-carrying regions in six strains with *L. petauri* L1 as the reference. Figure S4: Alignment of non-phosphotransferase system (NPTS) sucrose operon from *L. petauri* strain FDAARGOS _1063 (GenBank accession GCF_016128255.1) and *L. garvieae* strain BA06/02552-1 (GenBank accession GCF_030211605.1).

Table S1 to S6 spectrum.00101-25-s0002.xlsx Table S1: Details of 76 genomes from this work and 241 genomes from the NCBI included in the study. Table S2: Summary of findings for 76 isolates sequenced in this study. Table S3: Heatmap of pairwise SNP matrix for 76 genomes of *L. formosensis*, *L. garvieae,* and *L. petauri*. Table S4: Heatmap of pairwise SNP matrix for 53 *Lactococcus petauri* genomes. Table S5: Heatmap of pairwise comparison 191 phosphotransferase system (PTS) operons from 7 *Lactococcus* species. Table S6: Store and origins of food samples tested in this study.

ASM does not own the copyrights to Supplemental Material that may be linked to, or accessed through, an article. The authors have granted ASM a non-exclusive, world-wide license to publish the Supplemental Material files. Please contact the corresponding author directly for reuse.
