# Supplementary material for: Antimicrobial susceptibility and genomic characterization of Lactococcus formosensis, Lactococcus garvieae, and Lactococcus petauri in Hong Kong
Source: Microbiol Spectr. 2025 Aug 13;13(10):e00101-25. doi: 10.1128/spectrum.00101-25 (PMC12502582; doi:10.1128/spectrum.00101-25)
Supplement: Figure S1 to S4 — Figure S1: Visualization of MLST genetic clusters for L. petauri, L. formosensis, and L. garvieae from Hong Kong, 2021-2023. Figure S2: Alignment of LsaD amino acid sequences from 11 isolates. Figure S3: Comparative analysis of sucrose operon-carrying regions in six strains with L. petauri L1 as the reference. Figure S4: Alignment of non-phosphotransferase system (NPTS) sucrose operon from L. petauri strain FDAARGOS _1063 (GenBank accession GCF_016128255.1) and L. garvieae strain BA06/02552-1 (GenBank accession GCF_030211605.1). [file spectrum.00101-25-s0001.pdf]

Supplementary file 2

Figure S1 – Visualization of MLST genetic clusters for *L. petauri*, *L. formosensis*, and *L garvieae* from Hong Kong, 2021-2023. Each node represents one MLST sequence type. The isolate source is indicated by colors in the nodes. The species are indicated by color branches. The scale bar indicates distance for one allele difference.

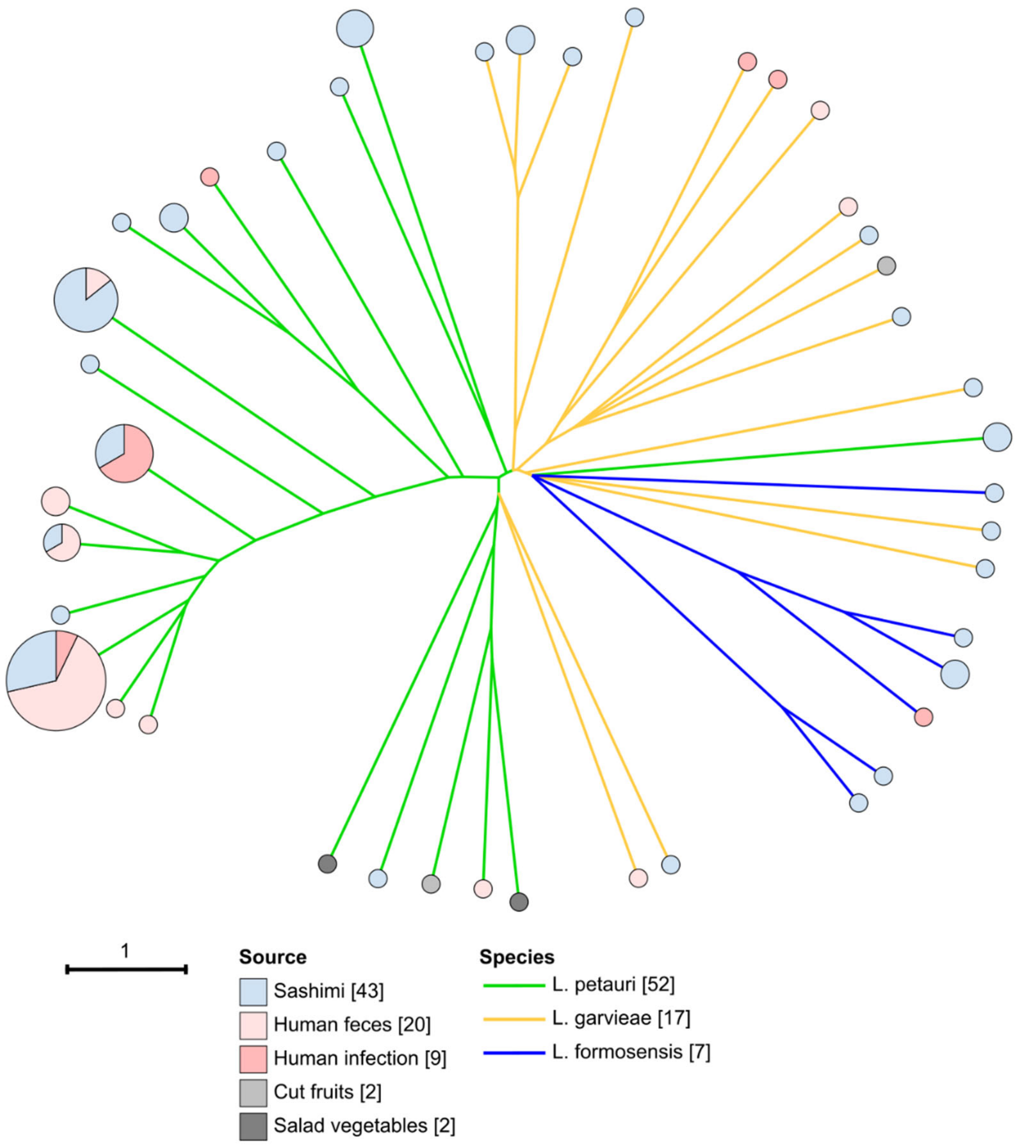

Figure S2. Alignment of LsaD amino acid sequences from 11 isolates. These include three reference sequences (L5, Sashimi-1038, and L1) associated with clindamycin resistant phenotype and eight sequences (Sashimi-1092, Sashimi-661, L2, L7, Fecal-478, Sashimi-1651, Fecal-587 and Fecal-622) associated with conversion to clindamycin susceptibility phenotypes. Key motifs were indicated in blue font and labeled. Insertion sequence disruption, premature stop, and substitutions R246H, L355P and D447Y were indicated in red font. Color shading showed the putative boundaries of nucleotide binding domain 1 (pink), interdomain linker region (blue) and nucleotide binding domain 2 (green) (1).

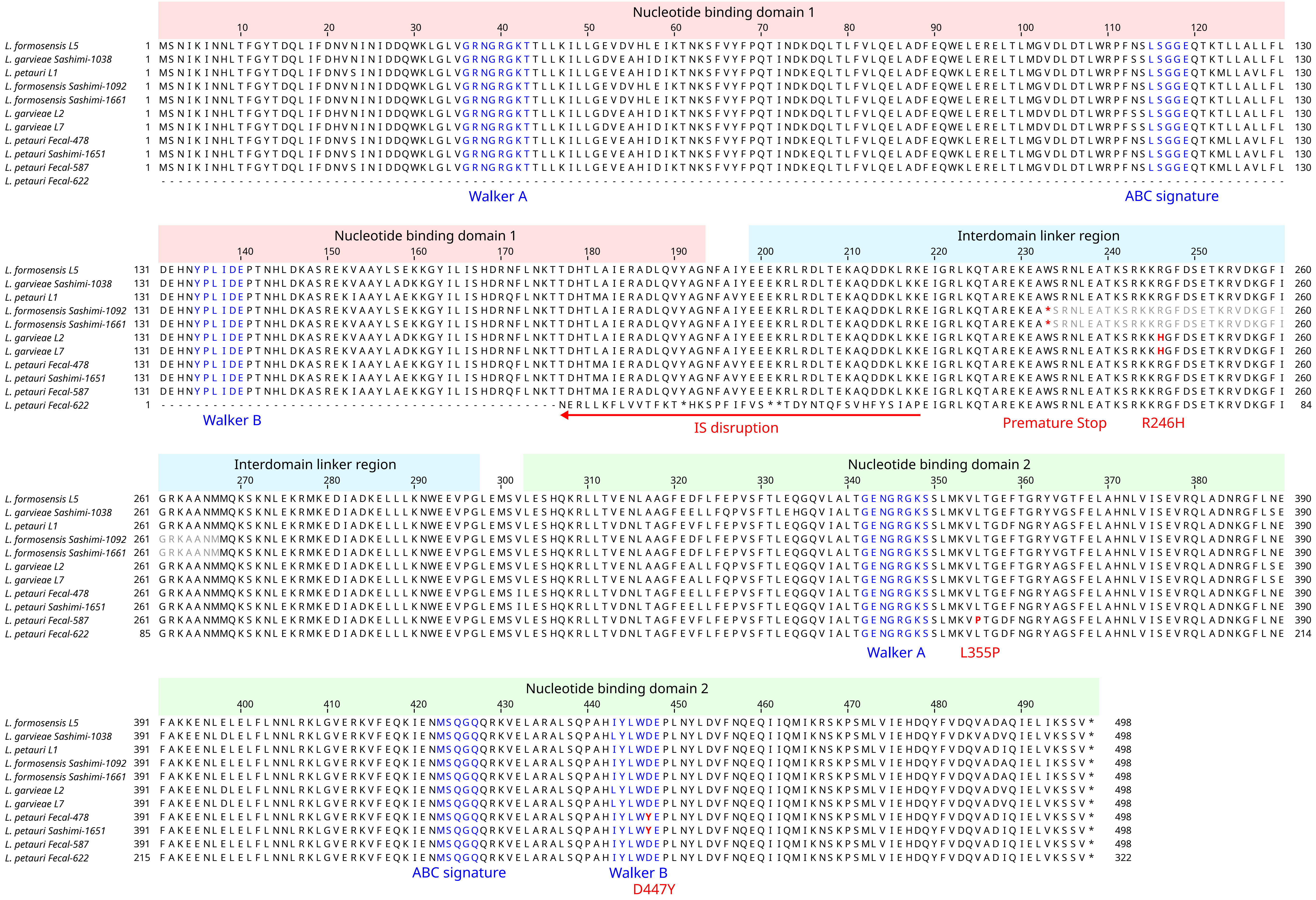
Nucleotide binding domain 1
Walker A
ABC signature
Interdomain linker region
Walker B
IS disruption
Premature Stop
R246H
Nucleotide binding domain 2
L355P
D447Y
L. formosensis L5
L. garvieae Sashimi-1038
L. petauri L1
L. formosensis Sashimi-1092
L. formosensis Sashimi-1661
L. garvieae L2
L. garvieae L7
L. petauri Fecal-478
L. petauri Sashimi-1651
L. petauri Fecal-587
L. petauri Fecal-622

Figure S3. Comparative analysis of sucrose operon-carrying regions in six strains with *L. petauri* L1 as the reference. The alignment illustrated presence of both PTS (Tn*5276*) and PTS (*L. petauri*) operons in three strains (Y20, Y35-2 and Y60-2) and the deletion of PTS (*L. petauri*) operon in 3 *L. petauri* strains: INF110 (GenBank accession GCF_018348575.1), 57 (GenBank accession GCF_030219765.1), BM06/00349 (GenBank accession GCF_030211135.1). PTS, phosphotransferase system

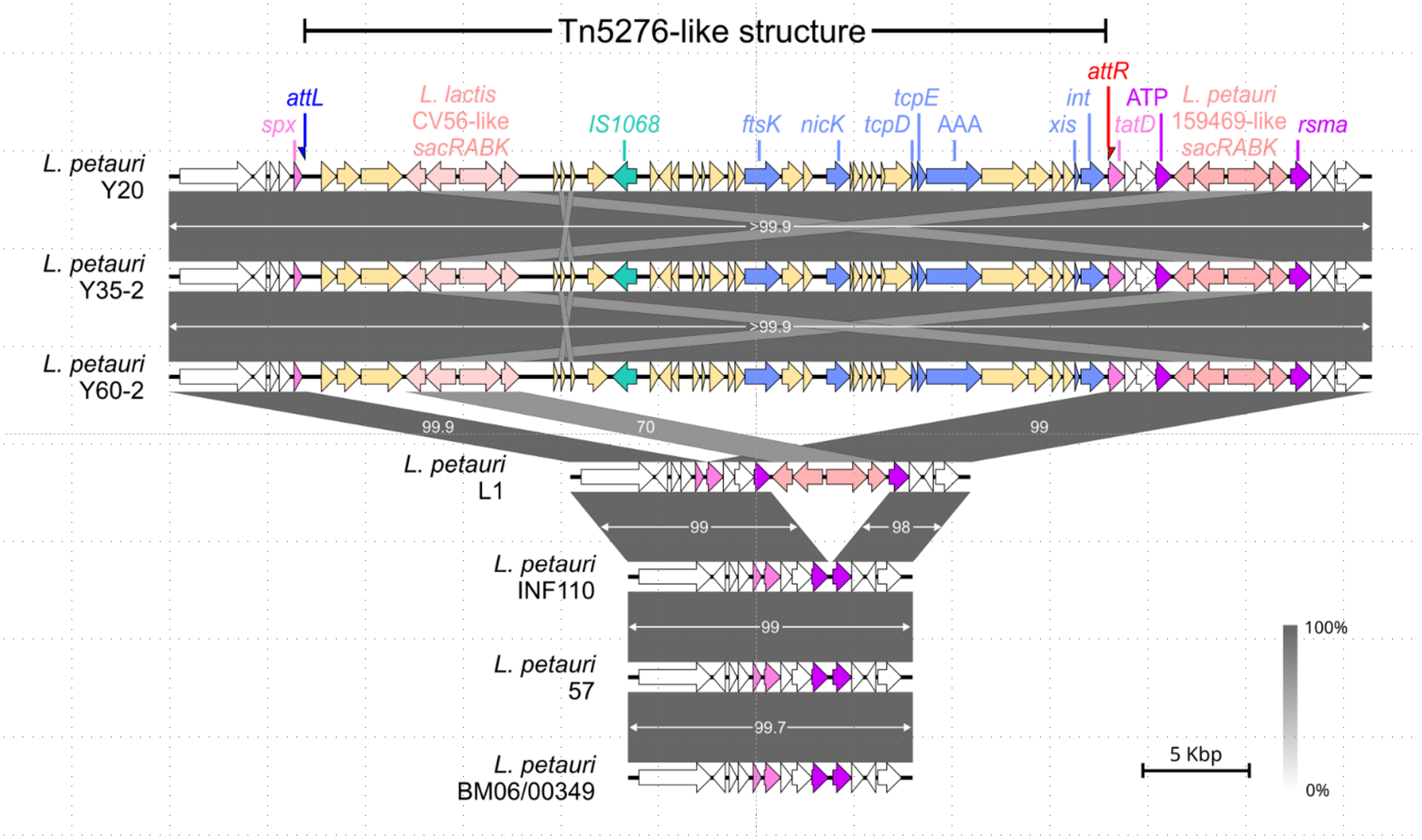

Figure S4. Alignment of non-phosphotransferase system (NPTS) sucrose operon from *L. petauri* strain FDAARGOS _1063 (GenBank accession GCF_016128255.1) and *L. garvieae* strain BA06/02552-1 (GenBank accession GCF_030211605.1). The genes in the NPTS sucrose operon are indicated in pink: *scrR*, sucrose operon repressor; *scrK*, fructokinase; *scrB*, sucrose-6-phosphate hydrolase, *lacY,* lactose permease. Insertion sequences (IS-*LL6*) and putative Tn*3* family transposase (*tnpR*) are indicated in green. CHAP, CHAP domain-containing protein gene; celC, lactose/cellobiose PTS EIIA component gene. In strain BA06/02552-1, the operon was detected in a contig of size 38.2 kb.

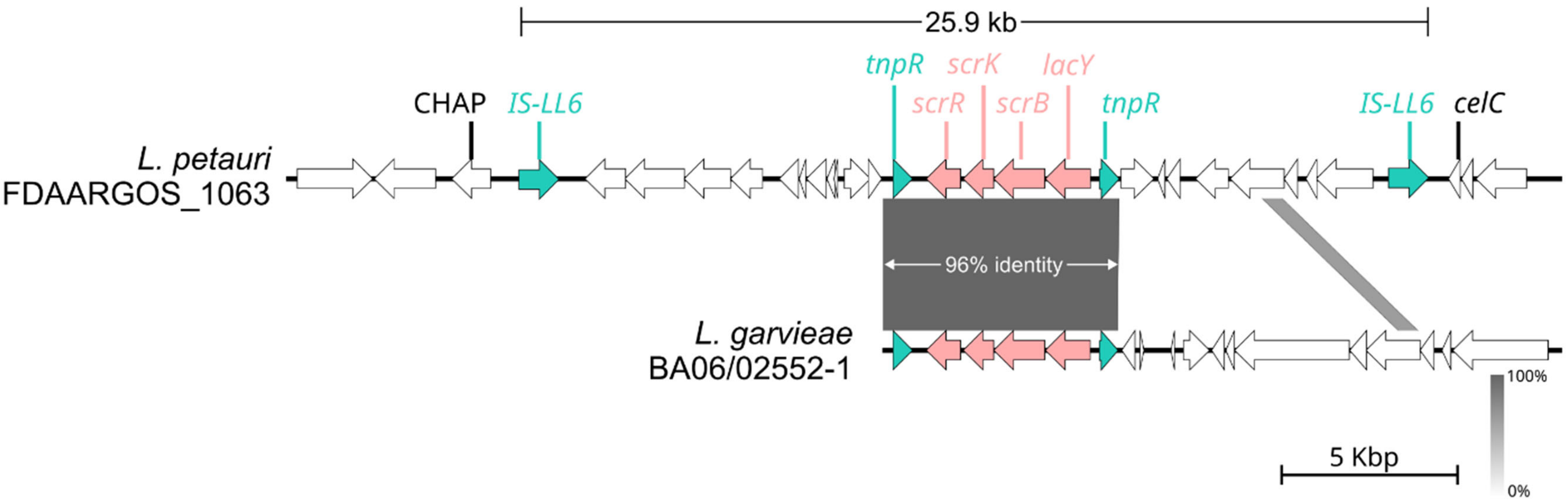

References

1. Crowe-McAuliffe C, Murina V, Turnbull KJ, Kasari M, Mohamad M, Polte C, Takada H, Vaitkevicius K, Johansson J, Ignatova Z, Atkinson GC, O'Neill AJ, Hauryliuk V, Wilson DN. 2021. Structural basis of ABCF-mediated resistance to pleuromutilin, lincosamide, and streptogramin A antibiotics in Gram-positive pathogens. Nat Commun 12:3577. doi:10.1038/s41467-021-23753-1 [pii];23753 [pii];10.1038/s41467-021-23753-1 [doi].
